# Prevalence and factors associated with high-risk human papillomavirus infection in women living with HIV/AIDS at a referral center: a cross-sectional study, Vitória, 2021-2022

**DOI:** 10.1590/S2237-96222025v34e20240796.en

**Published:** 2025-10-10

**Authors:** Waltesia Perini Rocha, Tania Reuter, Pâmela Cristina Gaspar, Francisco Naildo Cardoso Leitão, Giovanna Barille, Neide Aparecida Tosato Boldrini, Mariângela Freitas da Silveira, Angelica Espinosa Miranda

**Affiliations:** 1 Universidade Federal do Espírito Santo, Programa de Pós-Graduação em Saúde Coletiva, Vitória, ES, Brazil; 2 Hospital Universitário Cassiano Antonio de Moraes, Vitória, ES, Brazil; 3 Universidade Federal do Espírito Santo, Departamento de Clínica Médica, Vitória, ES, Brazil; 4 Ministério da Saúde, Departamento de HIV/Aids, Tuberculose, Hepatites Virais e Infecções Sexualmente Transmissíveis, Brasília, DF, Brazil; 5 Universidade Federal do Acre, Rio Branco, AC, Brazil; 6 Hospital Jayme dos Santos Neves, Serra, ES, Brazil; 7 Universidade Federal do Espírito Santo, Departamento de Ginecologia e Obstetrícia, Vitória, ES, Brazil; 8 Universidade Federal de Pelotas, Programa de Pós-Graduação em Epidemiologia, Pelotas, RS, Brazil

**Keywords:** Human Papillomavirus Viruses, HIV, Genotype, Squamous Intraepithelial Lesions of the Cervix, Cross-Sectional Studies, Virus del Papiloma Humano, VIH, Genotipo, Lesiones Intraepiteliales Escamosas de Cuello Uterino, Estudios Transversales

## Abstract

**Objective:**

To analyze the prevalence of high-risk human papillomavirus (HPV) infection in women living with HIV/AIDS and to identify associated risk factors and genotypic diversity by RT-PCR.

**Methods:**

This was a cross-sectional study of HIV-positive cisgender women treated at an HIV/AIDS referral center in Espírito Santo between May 2021 and May 2022. The study included women aged 18-64 on antiretroviral therapy with no cognitive or clinical deficits. For HPV detection and genotyping, vaginal self-collection was carried out for real-time polymerase chain reaction (RT-PCR). Sociodemographic, behavioral, laboratory, and clinical data were analyzed using Fisher’s exact and chi-square tests to verify associations with HPV. Poisson regression with robust variance was used to estimate prevalence ratios (PR) and 95% confidence intervals (95%CI). A p-value<0.05 was considered significant.

**Results:**

Of the 207 self-collected samples, 100.0% were valid for RT-PCR. HPV prevalence was 60.4%, with genotypes 58, 68, and 52 being the most common. In the adjusted analysis, detectable HIV/AIDS viral load was associated with a 37.0% increase in the probability of HPV infection (PR 1.37; 95%CI 1.00; 1.88). HPV-18 was associated with five times more alterations in cervical cytopathology.

**Conclusion:**

Women with HIV/AIDS had a high prevalence of high-risk HPV, predominantly genotype 58, with a higher probability of infection associated with detectable viral load. The validity of the self-collected samples proves the efficacy of the technique in HPV screening, reinforcing its potential in the prevention of cervical cancer.

## Resumen

**Objetivo:**

Analizar la prevalencia de la infección por el virus del papiloma humano (VPH) de alto riesgo en mujeres que viven con VIH/sida, así como identificar los factores de riesgo asociados y la diversidad genotípica mediante RT-PCR.

**Métodos:**

Estudio transversal realizado con mujeres cisgénero VIH positivas atendidas en un centro de referencia en VIH/sida en Espírito Santo, entre mayo de 2021 y mayo de 2022. Se incluyeron mujeres de 18 a 64 años en tratamiento antirretroviral, sin déficits cognitivos ni clínicos. Para la detección y genotipificación del VPH, se realizó autorrecolección vaginal para la reacción en cadena de la polimerasa en tiempo real (RT-PCR). Se analizaron datos sociodemográficos, conductuales, de laboratorio y clínicos utilizando la prueba exacta de Fisher y la prueba de chi-cuadrado para verificar asociaciones con el VPH. Se utilizó regresión de Poisson con varianza robusta para estimar razones de prevalencia (RP) e intervalos de confianza del 95 % (IC95 %). Se consideró significativo un valor de p < 0,05.

**Resultados:**

De las 207 muestras auto-recolectadas, el 100,0 % fueron válidas para RT-PCR. La prevalencia de VPH fue del 60,4 %, siendo los genotipos 58, 68 y 52 los más comunes. En el análisis ajustado, la carga viral detectable de VIH/sida se asoció con un aumento del 37,0 % en la probabilidad de infección por VPH (RP 1,37; IC95% 1,00; 1,88). El VPH-18 se asoció con un riesgo cinco veces mayor de alteraciones en la citopatología cervical.

**Conclusión:**

Las mujeres con VIH/sida presentaron una alta prevalencia de VPH de alto riesgo, con predominio del genotipo 58, y una mayor probabilidad de infección asociada a la carga viral detectable. La validez de las muestras autorrecolectadas demuestra la eficacia de la técnica en el tamizaje del VPH, lo que refuerza su potencial en la prevención del cáncer de cuello uterino.

**Ethical aspects**

This research respected ethical principles, having obtained the following approval data:

Research Ethics Committee: Hospital Universitário Cassiano Antonio de Moraes

Opinion number: 4,685,258

Approval date: 1/5/2021

Certificate of Submission for Ethical Appraisal: 43223521.7.2004.5071

Informed Consent Form: Obtained from all participants before data collection.

## Introduction

Persistent high-risk human papillomavirus (HPV) infection is the main cause of cervical cancer (1), a serious public health problem, especially in developing countries, where most deaths from this disease occur (2).

Among women living with HIV/AIDS, the presence of HPV is even more common, increasing the risk of developing cervical cancer and contributing to higher mortality rates in this group (3,4).

Despite advances in the treatment of HIV/AIDS infection with the use of antiretroviral therapy (ART), incidence of cervical cancer in this population remains high (5).

Different types of HPV can cause cervical cancer, especially types 16 and 18, which are responsible for the majority of cases (6). However, other types, such as types 31, 33, 52, and 58, are also important, especially in women with HIV/AIDS (7).

In Brazil, recent studies have shown that many women with HIV/AIDS have high-risk HPV infection, with differences in infection rates depending on the region of the country, which reinforces the need for more specific strategies to protect these women (8).

Due to the impact of HPV infection on the health of women living with HIV/AIDS, it is essential to identify the oncogenic HPV types early on and understand the factors that increase the risk of infection. This can help to improve preventive screenings, expand vaccinations, and treat cases early, reducing the chances of developing cervical cancer (9).

This study aimed to analyze the prevalence of high-risk HPV infection in women living with HIV/AIDS, identify the associated risk factors, and characterize the different HPV genotypes by RT-PCR.

## Methods

### Study design

This was a cross-sectional, descriptive, and analytical study carried out using data from the national survey “Detection of HPV DNA in vaginal samples self-collected by women living with HIV treated through the Brazilian public health system.” (10)

### Setting

The study was conducted at the infectious diseases outpatient clinic of the Cassiano Antonio Moraes University Hospital, Espírito Santo, from May 2021 to May 2022, during routine patient visits.

### Participants

We included cisgender women living with HIV/AIDS, aged 18-64, on antiretroviral therapy (ART), without cognitive or clinical deficits, with a history of at least one sexual relationship. Pregnant women, hysterectomized women, and women with a history of cervical cancer were excluded. A total of 207 participants were selected sequentially, according to routine visits to the infectious diseases outpatient clinic. 

### Variables

The dependent variable was the presence of high-risk HPV. To identify the types of HPV, vaginal self-collection was carried out, in which the participants received Coari self-collection kits (Kolplast, Brazil) with preservative fluid and an illustrated guide. Trained health professionals gave additional instructions using a pelvic model (Semina TM). Sample collection took place in a private place, unsupervised and unaccompanied. The self-collected samples were given to the health professional, identified with a standardized code, and stored at room temperature for up to 90 days until they were sent to the Molecular Biology, Microbiology, and Serology Laboratory at the Federal University of Santa Catarina by a specialized carrier. At the testing laboratory, DNA was extracted using the ReliaPrep Bloodg DNA Miniprep System kit (Promega, USA), and genotyping was carried out using the Anyplex II HPV28 Detection kit (Seegene, Korea), which detects 19 high-risk and nine low-risk genotypes, as well as an internal reaction control (endogenous human gene).

Amplification occurred in the CFX96 real-time thermal cycler (Bio-Rad, USA) and was analyzed using Seegene Viewer software. The results were sent electronically to our research center.

The study’s independent variables were sociodemographic, behavioral and clinical, collected through a semi-structured questionnaire administered in person by a trained interviewer, lasting an average of 20 minutes, and recorded on an online form, including the following variables: age, in complete years, categorized into age groups (18-29, 30-39, 40-49, 50-64 years); schooling, in years of study, categorized into four groups (0-4, 5-8, 9-11, 12 years or more); self-reported race/skin color (White, Brown, Black, Asian, Indigenous); marital status (single, married, widowed, separated); family income in minimum wages (≤1, between 1 and 3, and more than 3), smoking (never smoked, former smoker, current smoker), use of alcohol in the last four weeks (never, less than once a week, at least once a week, every day), use of illicit drugs (no, yes); history of pregnancy (0 to 4, 5 or more); age of first sexual intercourse (before the age of 15 or after); number of sexual partners throughout life (1 to 2, 3 to 4, 5 or more); sexual partners in the last 12 months (0, 1, 2 or more); HPV vaccination (no, yes); anal intercourse (no, yes); condom use (no, yes); diagnosis of sexually transmitted infection given by doctor (no, yes); and altered cervical cytopathology test (no, yes).

Laboratory data, such as HIV viral load, cluster of differentiation 4 (CD4) count, and use of ART, were extracted from medical records and checked by the national electronic system. Tests carried out up to six months before enrollment in the study were considered, with new tests for participants with older tests. Viral load was classified as undetectable at less than 20 copies/ml (real-time PCR, Abbott Real Time HIV-1). CD4 counts were taken by flow cytometry. Cervical cytopathology tests carried out up to a year before enrollment were accepted, and new tests were taken in cases where the tests were older. Colposcopy and biopsy were performed according to the cervical cytopathology abnormalities detected. The results of the RT-PCR for HPV were informed to the participants by telephone and recorded in their medical records. Participants with oncogenic genotypes were referred to the gynecology service at the Cassiano Antonio Moraes University Hospital for further screening and specialized treatment.

### Data sources/measurement

The sample calculation was based on the prevalence of high-risk HPV in women living with HIV/AIDS in Brazil, which was 28.4%, according to a multicenter study carried out previously (11). The calculated sample size was 201 women and, considering the loss of 20.0%, the expected sample size for the study was 241 women. A total of 246 participants were interviewed; 35 did not agree to participate in the study, and four did not meet the eligibility criteria.

### Statistical methods

The data was analyzed using STATA 16.1 software (Stata Corp). Absolute and relative frequencies were calculated for the descriptive variables, including the prevalence of high-risk and/or probable high-risk HPV, which were grouped for high-risk HPV analysis. The means and standard deviation (SD) were calculated for the continuous variables. Associations between high-risk HPV and independent variables were analyzed using Fisher’s exact test and chi-square tests. Variables with a p-value<0.20 in the crude analysis were included in the Poisson regression model with robust variance, estimating prevalence ratios (PR), and 95% confidence intervals (95%CI). A p-value<0.05 was considered statistically significant.

## Results

207 cisgender women (85.9%) participated in the study, and all the self-collected samples were valid for HPV testing (data not shown). The mean (SD) age was 48.7 (10.7) years. High-risk HPV infection was recorded in 125 samples (60.4%). Concerning sociodemographic and behavioral variables (Table 1), the highest prevalence of HPV was among women aged 18-29 (78.6%), with a family income of between 1 and 3 minimum wages (69.5%) and who used condoms (68.5%).

**Table 1:** Prevalence (P) and 95% confidence intervals (95%CI) of high-risk HPV in women living with HIV/AIDS, according to sociodemographic, behavioral, and clinical characteristics. Vitória, May 2021 to May 2022 (n=207)

Variable	n (%)	High-risk HPV	
		P (95%CI)	p-value^a^
**Age group** (years)			0.047
18-29	14 (6.8)	78.6 (50.4; 93.0)	
30-39	38 (18.4)	76.3 (60.3; 87.2)	
40-49	64 (30.9)	54.7 (42.4; 66.4)	
50-64	91 (44.0)	54.9 (44.6; 64.9)	
**Schooling** (years)			0.914
0-4	43 (20.8)	55.8 (7.6; 69.8)	
5-8	53 (25.6)	62.3 (48.6; 74.3)	
9-11	92 (44.4)	60.9 (50.5; 70.3)	
≥12	19 (9.2)	63.2 (40.2; 81.4)	
**Race/skin color**			0.460
White	66 (31.9)	60.6 (48.4; 71.7)	
Brown	100 (48.3)	57.0 (47.1; 66.4)	
Black	41 (19.8)	68.3 (52.6; 80.7)	
**Marital status**			0.871
Single	35 (16.9)	57.1 (40.5; 72.3)	
Widowed	40 (19.3)	60.0 (44.3; 73.9)	
Married	91 (44.0)	59.3 (48.9; 69.0)	
Separated	41 (19.8)	65.9 (50.2; 78.7)	
**Family income** (**minimum wage**)			0.039
≤1	80 (42.1)	53.8 (42.8; 64.4)	
>1 ≤3	82 (43.2)	69.5 (58.7; 78.5)	
>3	28 (14.7)	46.4 (29.1; 64.7)	
**Smoking**			0.612
Never smoked	114 (55.3)	63.2 (53.9; 71.5)	
Former smoker	61 (29.6)	55.7 (43.1; 67.7)	
Current smoker	31 (15.1)	58.1 (40.3; 73.9)	
**Use of alcohol in the last month**			0.220
Never	115 (55.8)	40.0 (53.4; 71.0)	
Less than once a week	49 (23.8)	49.0 (35.3; 62.8)	
At least once a week	37 (18.0)	70.3 (53.8; 82.8)	
Every day	5 (2.4)	60.0 (19.9; 90.1)	
**Uses illicit drugs**			0.219
No	178 (86.0)	58.4 (51.0; 65.5)	
Yes	29 (14.0)	72.4 (53.6; 85.6)	
**Pregnancy history**			0.960
No	23 (11.1)	60.9 (40.1; 78.3)	
Yes	184 (88.9)	60.3 (53.0; 67.2)	
**Number of pregnancies by groups**			0.658
0 to 4	183 (88.4)	59.6 (52.3; 66.5)	
5 or more	24 (11.6)	66.7 (46.0; 82.4)	
**Age of first sexual intercourse** (years)			0.298
<15	44 (21.4)	58.6 (50.9; 66.0)	
≥15	162 (78.6)	68.2 (53.1; 80.2)	
**Sexual partners in life**			0.585
1-2	61 (29.5)	57.4 (44.7; 69.2)	
3-4	56 (27.1)	66.1 (52.7; 77.3)	
≥5	90 (43.5)	58.9 (48.4; 68.6)	
**Sexual partners in the last 12 months**			0.737
0	62 (30.2)	58.1 (45.5; 69.7)	
1	127 (62.0)	60.6 (51.8; 68.8)	
≥2	16 (7.8)	68.8 (43.2; 86.4)	
**HPV vaccine**			0.767
No	195 (94.2)	60.0 (52.9; 66.7)	
Yes	12 (5.8)	66.7 (37.4; 87.0)	
**Anal intercourse**			0.885
No	122 (59.2)	61.5 (52.5; 69.7)	
Yes	84 (40.8)	59.5 (48.7; 69.5)	
**Condom use**			0.045
No	115 (55.6)	53.9 (44.7; 62.8)	
Yes	92 (44.4)	68.5 (58.3; 77.2)	
**Abnormal cervical cytology**			0.220
No	190 (91.8)	58.9 (51.8; 65.8)	
Yes	17 (8.2)	76.5 (51.3; 90.9)	
CD4^b^			0.858
≤499	40 (19.3)	59.9 (52.2; 67.1)	
≥500	167 (80.7)	62.5 (46.7; 76.0)	
**HIV viral load**			
<20 undetectable	190 (91.8)	58.4 (51.2; 65.3)	0.070
≥20 detectable	17 (8.2)	82.4 (57.1; 94.2)	
**Currently using a protease inhibitor**			0.669
No	94 (45.4)	58.5 (48.3; 68.1)	
Yes	113 (54.6)	61.9 (52.6; 70.5)	
**Time on antiretroviral therapy** (years)			0.220
0 to 4	28 (13.5)	58.7 (51.3; 65.7)	
>4	179 (86.5)	71.4 (52.3; 85.1)	
**Sexually transmitted infection diagnosed by a doctor^c^**			
No	179 (92.7)	60.9 (53.5; 67.8)	0.802
Yes	14 (7.3)	64.3 (37.5; 84.4)	

^a^p-value corresponds to Fisher’s exact test for dichotomous variables and the chi-square test for polytomous variables; ^b^CD4: cluster of differentiation 4; ^c^Diagnosis of sexually transmitted infection given by a doctor is the variable with the highest number of missing data (7%).

Concerning clinical factors (Table 1), the highest prevalence of high-risk HPV was found in participants with altered cervical cytopathology (76.5%, 95%CI 51.3; 90.9) and detectable HIV/AIDS viral load ≥20 copies/mL (82.4%, 95%CI 57.1; 94.2). 

In the adjusted analysis (Table 2), only the HIV/AIDS viral load remained significantly associated with high-risk HPV infection. Women with a detectable viral load (≥20 copies/mL) had a 37.0% higher prevalence of high-risk HPV infection compared to those with an undetectable viral load (<20 copies/mL) (PR 1.37; 95%CI 1.00; 1.88; p-value 0.046). The other variables (age group, family income, and condom use) showed association trends but without statistical significance within the 95%CI. 

**Table 2:** Adjusted prevalence ratios (PR) and 95% confidence intervals (95%CI) for high-risk HPV associations in women living with HIV/AIDS with valid tests. Vitória, May 2021 to May 2022 (n=207)

Variable	High-risk HPV	p-value^a^
	PR (95%CI)	
**Age group** (years)		0.332
18-29	1.18 (0.78; 1.80)	
30-39	1.26 (0.93; 1.69)	
40-49	0.97 (0.72; 1.30)	
50-64	Ref.	
**Family income** (**minimum wage**)		0.071
≤1	1.10 (0.70; 1.72)	
>1 ≤3	1.40 (0.72; 1.30)	
>3	Ref.	
**Condom use**		0.068
No	Ref.	
Yes	1.24 (0.98; 1.56)	
**HIV viral load**		0.046
<20 undetectable	Ref.	
≥20 detectable	1.37 (1.00; 1.88)	

^a^The p-values correspond to the z-test of the Poisson regression with robust variance.

**Figure 1:**
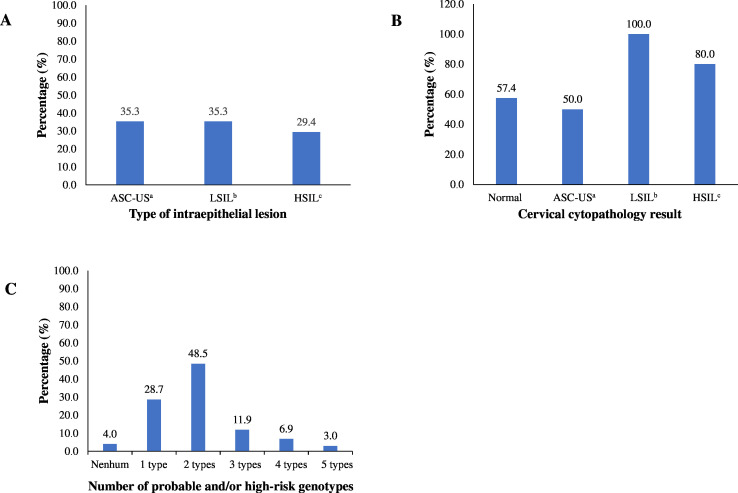
Distribution of cervical cytopathology abnormalities (n=17) (A); presence of high-risk HPV according to cytopathology results (n=207) (B); and number of high-risk HPV genotypes in cases of multiple infection (n=101) (C) among women with HPV living with HIV/AIDS. Vitória, May 2021 to May 2022

When analyzing cervical cytopathology prevalence, abnormalities accounted for 8.2% (17/207). Among these, 29.4% (5/17) corresponded to high-grade intraepithelial lesions, 35.3% (6/17) to low-grade intraepithelial lesions, and 35.3% (6/17) to atypical squamous cells of undetermined significance (Figure 1A). Among the samples with normal cervical cytopathology (190/207), 57.4% tested positive for high-risk HPV types. In the samples with altered cervical cytopathology, high-risk HPV was detected in 100.0% of low-grade intraepithelial lesions, 50.0% of atypical squamous cells of undetermined significance, and 80.0% of high-grade intraepithelial lesions (Figure 1B). Altered cervical cytopathologies were observed in three patients with undetectable HPV, one with a high-grade intraepithelial lesion, and two with atypical squamous cells of undetermined significance. Almost half (45.5%) of the women with multiple genotypes had two high-risk HPV types, while 28.7% had only one type (Figure 1C).

**Figure 2:**
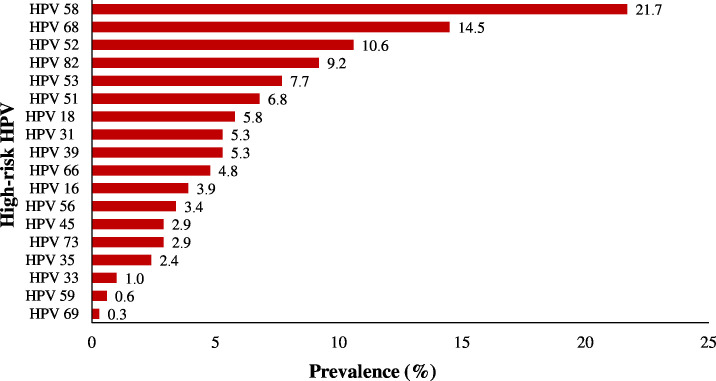
Distribution of high-risk HPV types in women living with HIV/AIDS. Vitória, May 2021 to May 2022 (n=207)

Analysis of the distribution of high-risk HPV types (Figure 2) revealed that type 58 was the most prevalent, present in 21.7% of the samples, followed by HPV 68 (14.5%) and HPV 52 (10.6%). HPV types 16 and 18 were found in 3.9% and 5.8% of the samples.

Analysis of association between HIV/AIDS status and HPV with cytological abnormalities (Table 3) showed that women with HPV 18 infection were five times more likely to have cervical cytopathology abnormalities, with a prevalence of 33.3% (PR 5.0; 95%CI 1.9; 13.1; p-value 0.001), this being a statistically significant association. This result suggests that HPV 18 is an important risk factor for cytological abnormalities. HPV 58 infection was also associated with an increased risk of cytological alterations (PR 2.5; 95%CI 1.0; 6.3; p-value 0.05), although the results indicate a trend of association that the sample size may have influenced. No association was found with HPV 16. Other variables were not associated with altered cervical cytopathology.

**Table 3:** Prevalence ratios (PR) and confidence intervals (95%CI) for the association between HIV/AIDS status and HPV with cytopathologic abnormalities. Vitoria, May 2021 to May 2022

Variables	Abnormal cervical cytology		p-value^a^
	Prevalence (%)	PR (95%CI)	
CD4^b^			0.856
≤499	8.4	Ref.	
≥500	7.5	0.9(0.3; 3.0)	
**HIV viral load**			0.136
<20	7.4	Ref.	
≥20	17.7	2.4(0.8; 7.5)	
**Currently using a protease inhibitor**			0.179
No	5.3	Ref.	
Yes	10.6	2.0(0.7; 5.5)	
**Time on antiretroviral therapy** (years)			0.207
0to4	14.3	2.0(0.7; 5.6)	
>4	7.3	Ref.	
HPV16			0.648
No	8.0	Ref.	
Yes	12.5	1.6(0.2; 10.4)	
HPV18			0.001
No	6.7	Ref.	
Yes	33.3	5.0(1.9; 13.1)	
HPV58			0.050
No	6.2	Ref.	
Yes	15.6	2.5(1.0; 6.3)	
**Multiple genotypes**			0.073
No	4.7	Ref.	
Yes	11.9	2.5(0.9; 6.9)	

^a^p-value Waldtest; ^b^CD4: cluster of differentiation 4.

## Discussion

The findings of this study indicate that women living with HIV/AIDS have a high prevalence of high-risk HPV infection, especially genotypes not included in the vaccine currently available through the Brazilian National Health System. This susceptibility was associated with detectable HIV viral load, demonstrating the importance of using ART to maintain HIV viral load suppressed in order to reduce the prevalence of high-risk HPV. The results reinforce that the full validation of samples obtained by vaginal self-collection for the PCR-HPV test demonstrates the efficacy and viability of this strategy as a primary screening method, contributing both to the early diagnosis of pre-neoplastic lesions of the uterine cervix and to the identification of high-risk HPV genotypes.

One of the limitations of this study was its cross-sectional design, which makes it impossible to establish causal relationships between the variables analyzed. In addition, there may be information bias related to exposure to certain risk factors, such as the use of condoms and the number of sexual partners, since these data were obtained through self-reporting. The diagnostic approach also has restrictions since it was based exclusively on cervical cytopathology carried out in the last year, and colposcopy was only indicated in cases with cytological alterations. This strategy may have led to underestimating lesions in women with normal cytology tests. In turn, laboratory data quality was improved by checking the CD4+ T-lymphocyte count and HIV viral load records held on the national electronic system and checking vaccination cards individually.

The high prevalence of high-risk HPV infection observed in the study corroborates the findings of the national study carried out in 2023 (10), which reported 53.8% prevalence, ranging from 37.0% in São Paulo to 67.3% in Manaus and other regional studies (12-14). Globally, prevalence of oncogenic HPV in women living with HIV/AIDS with normal cervical cytopathology is 48.4%, in contrast to 17.0% in seronegative women, which reflects the negative impact of HIV/AIDS infection on HPV infection (15). A systematic review carried out in 2020 (16) reported that on average prevalence of oncogenic HPV was 51.0% in developing countries, with significant variations: Benin had 87.5% prevalence (17), while in the Bahamas it was 78.0% (18). These high prevalence rates may reflect social inequalities in access to health care and higher prevalence of HIV/AIDS immunosuppression in these regions (16,19,20). Lower prevalence rates were observed in India (34.7%) (21) and Ethiopia (35.2%) (22), which may be related to local factors such as variations in the sociodemographic profile and public health strategies.

Immunosuppression resulting from HIV infection compromises the cellular immune response, hindering HPV clearance, favoring reactivation of latent infections, and contributing to viral persistence (23,24). In this study, detectable HIV viral load was identified as the main risk factor associated with oncogenic HPV infection, increasing the likelihood of infection by up to 37.0%. This finding is in line with evidence from similar populations in Ghana (25), Ethiopia (22), and Bahia (26). This association may be related to low patient adherence to ART, which results in a detectable viral load being maintained and favors the expression of HPV E6 and E7 oncoproteins. This process may be mediated by the action of HIV Tat proteins, which induce the release of chemokines capable of exacerbating the oncogenic activity of HPV (7).

Therefore, it is clear that although ART is effective in suppressing HIV viral load and reducing the prevalence of high-risk HPV, its impact is significantly enhanced when started early, preferably with a CD4 count above 500 cells/mm^3^, and maintained with strict adherence (27). Studies conducted in the Bahamas (18) and in the Brazilian Amazon region (28) reinforce this observation by identifying a higher prevalence of HPV infection in women with more severe immunosuppression, characterized by a CD4 count of less than 200 cells/mm^3^. These data reinforce the importance of early diagnosis of HIV with immediate treatment and continuous adherence to ART as important measures for mitigating the persistence of HPV and its associated complications.

Identifying high-risk HPV genotypes is essential for improving primary and secondary prevention measures in order to reduce the incidence and mortality of HPV-related cancers and strengthen vaccination programs. RT-PCR testing for HPV as a primary screening method for cervical cancer is crucial for identifying these high-risk genotypes. In this study, all self-collected samples were considered valid for the RT-PCR test for HPV, which aligns with a Brazilian study published in 2023 (10).

The prevalence of HPV types other than 16 and 18 is higher in the HIV/AIDS population when compared to their counterparts (15-18,21). In this study, type 58 was the most prevalent (21.7%), followed by types 68 (14.5%) and 52 (10.6%). Similar results were found in Rio de Janeiro (12), West Africa (17), and Tanzania (29). Also, surveys in Latin America and the Caribbean (30) and China in 2023 (31) identified type 58 as the second most prevalent. In China, type 58 was the third leading cause of cervical cancer.

In Brazil, high prevalence of oncogenic HPV other than 16 and 18 has been described (10,12), which is aligned with our findings. However, previous studies in Espírito Santo identified type 16 as the most prevalent, followed by other oncogenic types such as 33, 31, and 51 (14,32).

Although HPV 18 was not the most prevalent genotype in this sample, it was associated with a five times greater likelihood of cervical cytopathology alterations, highlighting its high oncogenic capacity (15). However, subtype 58 showed a tendency of being associated with cytological abnormalities, which corroborates the findings found in China in 2023 (31) and highlights the importance of monitoring genotypes other than 16 and 18 in women living with HIV/AIDS. This approach can support more comprehensive and effective prevention strategies to target intervention measures in this vulnerable population. The limited sample power of this study can explain the lack of association with HPV 16 in this analysis.

Despite the efficacy of the 4-valent vaccine against HPV types 16,^18^, 6, and 11, other oncogenic genotypes, such as HPV 58 and 52, are emerging, especially in immunocompromised populations (15,31). The population in this study showed low vaccination coverage (5.8%), but with 100.0% efficacy against the target types of the 4-valent vaccine used. However, 75% of those vaccinated had other oncogenic types, such as HPV 58, 52, 68, and 82, which highlights the need for continuous surveillance and possibly vaccines with greater genotypic coverage, as observed by studies in Brazil (33,34) and in other countries (31).

The study revealed a high prevalence of high-risk HPV in cisgender women living with HIV/AIDS, with a predominance of genotype 58 and an association with detectable HIV viral load. Vaginal self-collection, analyzed by RT-PCR, showed high validity and stands out as an effective strategy for HPV screening in public health. The findings reinforce the need for integrated actions, including early initiation and sustained adherence to ART, the expansion of screening with primary testing for HPV, and the use of the 9-valent vaccine in public immunization programs to reduce the burden of HPV/HIV co-infection and its complications.
