# Supplementary material for: Prevalence and factors associated with high-risk human papillomavirus infection in women living with HIV/AIDS at a referral center: a cross-sectional study, Vitória, 2021-2022
Source: Epidemiol Serv Saude. 2025 Oct 10;34:e20240796. doi: 10.1590/S2237-96222025v34e20240796.en (PMC12513468; doi:10.1590/S2237-96222025v34e20240796.en)
Supplement: Translation

# Translation: Prevalência e fatores associados à infecção pelo papilomavirus humano de alto risco em mulheres vivendo com HIV/aids em um centro de referência: estudo transversal, Vitória, 2021-2022

**DOI:** 10.1590/S2237-96222025v34e20240796.pt

**Authors:** Waltesia Perini Rocha, Tania Reuter, Pâmela Cristina Gaspar, Francisco Naildo Cardoso Leitão, Giovanna Barille, Neide Aparecida Tosato Boldrini, Mariângela Freitas da Silveira, Angelica Espinosa Miranda

**Affiliations:** 1 Universidade Federal do Espírito Santo, Programa de Pós-Graduação em Saúde Coletiva, Vitória, ES, Brasil; 2 Hospital Universitário Cassiano Antonio de Moraes, Vitória, ES, Brasil; 3 Universidade Federal do Espírito Santo, Departamento de Clínica Médica, Vitória, ES, Brasil; 4 Ministério da Saúde, Departamento de HIV/Aids, Tuberculose, Hepatites Virais e Infecções Sexualmente Transmissíveis, Brasília, DF, Brasil; 5 Universidade Federal do Acre, Rio Branco, AC, Brasil; 6 Hospital Jayme dos Santos Neves, Serra, ES, Brasil; 7 Universidade Federal do Espírito Santo, Departamento de Ginecologia e Obstetrícia, Vitória, ES, Brasil; 8 Universidade Federal de Pelotas, Programa de Pós-Graduação em Epidemiologia, Pelotas, RS, Brasil

**Keywords:** Papillomavirus Humano, HIV, Genótipos, Lesões Intraepiteliais Escamosas Cervicais, Estudos Transversais

## Abstract

**Objetivos:**

Analisar a prevalência de infecção pelo papilomavírus humano (HPV) de alto risco em mulheres vivendo com HIV/aids e identificar fatores de risco associados e diversidade genotípica por RT-PCR.

**Método:**

Tratou-se de estudo transversal com mulheres cisgênero HIV-positivas, atendidas em centro de referência para HIV/aids no Espírito Santo, entre maio de 2021 e maio de 2022. Foram incluídas mulheres entre 18-64 anos, em terapia antirretroviral, sem déficits cognitivos ou clínicos. Para detecção e genotipagem do HPV, realizou-se autocoleta vaginal para reação de cadeia em polimerase em tempo real (RT-PCR). Dados sociodemográficos, comportamentais, laboratoriais e clínicos foram analisados por meio dos testes exato de Fisher e qui-quadrado para verificar as associações com HPV. Para estimar as razões de prevalência (RP) e intervalos de confiança de 95% (IC95%), utilizou-se regressão de Poisson com variância robusta. Foi considerado significativo um p-valor<0,05.

**Resultados:**

Das 207 amostras autocoletadas, 100,0% foram válidas para RT-PCR. A prevalência do HPV foi de 60,4%, com os genótipos 58, 68 e 52 sendo os mais comuns. Na análise ajustada, a carga viral detectável do HIV/aids esteve associada ao aumento de 37,0% na probabilidade de infecção por HPV (RP 1,37; IC95% 1,00; 1,88). O HPV-18 esteve associado a cinco vezes mais alterações na citopatologia cervical.

**Conclusão:**

Mulheres com HIV/aids apresentaram alta prevalência de HPV de alto risco, predominando o genótipo 58, com maior probabilidade de infecção associada à carga viral detectável. A validade das amostras autocoletadas comprova a eficácia da técnica no rastreamento do HPV, reforçando seu potencial na prevenção do câncer cervical.

**Aspectos éticos**

A presente pesquisa respeitou os princípios éticos, obtendo os seguintes dados de aprovação:

Comitê de ética em pesquisa: Hospital Universitário Cassiano Antonio de Moraes

Número do parecer: 4.685.258

Data de aprovação: 1/5/2021

Certificado de apresentação de apreciação ética: 43223521.7.2004.5071

Registro do consentimento livre e esclarecido: Obtido de todos os participantes antes da coleta.

## Introdução

A infecção persistente pelo papilomavírus humano (HPV) de alto risco é a principal causa do câncer de colo do útero (1), um problema grave de saúde pública, principalmente em países em desenvolvimento, onde a maioria das mortes por essa doença acontece (2).

Entre mulheres que vivem com HIV/aids, a presença do HPV é ainda mais comum, aumentando o risco de desenvolvimento do câncer de colo do útero e contribuindo para taxas mais altas de mortalidade nesse grupo (3,4).

Apesar dos avanços no tratamento da infecção pelo HIV/aids com o uso da terapia antirretroviral (TARV), a incidência do câncer de colo do útero nessa população permanece elevada (5).

Diferentes tipos de HPV podem causar o câncer de colo do útero, com destaque para os tipos 16 e 18, que são responsáveis pela maioria dos casos (6). No entanto, outros tipos, como os dos tipos 31, 33, 52 e 58, também são importantes, especialmente em mulheres com HIV/aids (7).

No Brasil, estudos recentes mostraram que muitas mulheres com HIV/aids têm infecção pelo HPV de alto risco, com diferenças nas taxas de infecção dependendo da região do país, o que reforça a necessidade de estratégias mais específicas para proteger essas mulheres (8).

Devido ao impacto da infecção pelo HPV na saúde de mulheres vivendo com HIV/aids, é fundamental identificar precocemente os tipos oncogênicos do vírus e entender os fatores que aumentam o risco de infecção. Isso pode ajudar a melhorar os exames preventivos, ampliar a vacinação e tratar os casos precocemente, reduzindo as chances de desenvolvimento do câncer de colo uterino (9).

Este estudo teve como objetivo analisar a prevalência da infecção pelo HPV de alto risco em mulheres vivendo com HIV/aids, identificar os fatores de riscos associados e caracterizar os diferentes genótipos do HPV por RT-PCR.

## Métodos

### Desenho do estudo

Tratou-se de estudo transversal, descritivo e analítico realizado com os dados da pesquisa nacional “Detecção do DNA do HPV vaginal por autocoleta em mulheres vivendo com HIV/aids tratadas pelo sistema de saúde público brasileiro” (10).

### Local do estudo

O estudo foi conduzido no ambulatório de infectologia do Hospital Universitário Cassiano Antonio Moraes, Espírito Santo, no período de maio de 2021 a maio de 2022, durante visitas de rotina das pacientes.

### Critérios de elegibilidade

Foram incluídas mulheres cisgênero vivendo com HIV/aids, de 18-64 anos, usuárias de TARV, sem déficits cognitivos ou clínicos, com histórico de pelo menos uma relação sexual. Gestantes, histerectomizadas e mulheres com histórico de câncer cervical foram excluídas. Foram selecionadas 207 participantes de forma sequencial, conforme visitas de rotina no ambulatório de infectologia. 

### Variáveis

A variável dependente foi a presença do HPV de alto risco. Para identificar os tipos de HPV, foi realizada a autocoleta vaginal, em que as participantes receberam kits de autocoleta Coari (Kolplast, Brasil) com líquido conservante e um guia ilustrado. Orientações adicionais foram dadas por profissionais de saúde treinados utilizando um modelo pélvico (Semina TM). A coleta foi feita em local privado, sem supervisão e sem acompanhante. As amostras auto coletadas foram entregues ao profissional de saúde, identificadas com um código padronizado e armazenadas em temperatura ambiente por até 90 dias, até serem enviadas ao Laboratório de Biologia Molecular, Microbiologia e Sorologia da Universidade Federal de Santa Catarina, por transportadora especializada. No laboratório executor, o DNA foi extraído com o kit Relia Prep Bloodg DNA Miniprep System (Promega, EUA) e a genotipagem foi feita com o kit Anyplex II HPV28 Detection (Seegene, Coreia), que detecta 19 genótipos de alto risco e nove de baixo risco, além de um controle interno da reação (gene humano endógeno).

A amplificação ocorreu no termociclador CFX96 real-time (Bio-Rad, EUA) e foi analisada com uso do software Seegene Viewer. Os resultados foram enviados eletronicamente ao nosso centro de pesquisa.

As variáveis independentes do estudo foram as sociodemográficas, comportamentais e clínicas, coletadas por meio de um questionário semiestruturado aplicado presencialmente por um entrevistador treinado, com duração média de 20 minutos, e registrado em um formulário online, incluindo as seguintes variáveis: idade, em anos completos, categorizada em faixa etária (18-29, 30-39, 40-49, 50-64 anos); escolaridade, em anos de estudo, categorizada em quatro grupos (0-4, 5-8, 9-11, 12 anos ou mais); raça/cor da pele autorreferida (branca, parda, preta, amarela, indígena); estado civil (solteira, casada, viúva, separada); renda familiar em salários mínimos (≤1, entre 1 e 3, e mais de 3), tabagismo (nunca fumou, ex-fumante, fumante atual), uso de álcool nas últimas quatro semanas (nunca, menos de uma vez por semana, pelo menos uma vez por semana, todos os dias), uso de drogas ilícitas (não, sim); histórico de gravidez (0 a 4, 5 ou mais); idade da primeira relação sexual (antes dos 15 anos de idade ou depois); número de parceiros sexuais ao longo da vida (1 a 2, 3 a 4, 5 ou mais); parceiros sexuais nos últimos 12 meses (0, 1, 2 ou mais); vacina de HPV (não, sim); relação sexual anal (não, sim); uso de preservativo (não, sim); diagnóstico de infecção sexualmente transmissível dado pelo médico (não, sim); e exame citopatológico cervical alterado (não, sim).

Dados laboratoriais, como carga viral do HIV, contagem de cluster de diferenciação 4 (CD4) e uso de TARV, foram extraídos dos prontuários médicos e conferidos pelo sistema eletrônico nacional. Foram considerados exames realizados até seis meses antes da inscrição no estudo, com novas coletas para aquelas participantes com exames mais antigos. A carga viral foi classificada como indetectável quando menor que 20 cópias/mL (PCR em tempo real, Abbott Real Time HIV-1). A contagem de CD4 foi realizada por citometria de fluxo. Foram aceitos exames citopatológicos cervicais realizados até um ano antes da inscrição, sendo feitas novas coletas para os casos em que os exames fossem mais antigos. Colposcopia e biópsia foram realizadas conforme anormalidades citopatológicas cervicais detectadas. Os resultados do RT-PCR para HPV foram informados às participantes por telefone e registrados nos prontuários médicos. As participantes com genótipos oncogênicos foram encaminhadas para o serviço de ginecologia do Hospital Universitário Cassiano Antonio Moraes, para dar continuidade ao rastreamento e tratamento especializado.

### Cálculo amostral

O cálculo amostral foi baseado no dado de prevalência de HPV de alto risco em mulheres vivendo com HIV/aids no Brasil, que foi de 28,4%, conforme estudo multicêntrico realizado anteriormente (11). O tamanho da amostra calculado foi de 201 mulheres e, considerando-se a perda de 20,0%, o tamanho da amostra esperada para o estudo foi de 241 mulheres. Foram entrevistadas 246 participantes, 35 não aceitaram participar do estudo e quatro não atenderam aos critérios de elegibilidade.

### Análise estatística

Os dados foram analisados por meio do software STATA 16.1 (Stata Corp). Frequências absolutas e relativas foram calculadas para as variáveis descritivas, incluindo a prevalência de HPV de alto risco e/ou provável alto risco, que foram agrupadas para fins de análise de HPV de alto risco. Para as variáveis contínuas, foram calculadas as médias e o desvio-padrão (DP). Associações entre HPV de alto risco e variáveis independentes foram analisadas usando-se o teste exato de Fisher e o teste qui-quadrado. As variáveis com p-valor<0,20 na análise bruta foram incluídas no modelo de regressão de Poisson com variância robusta, estimando-se razões de prevalência (RP) e intervalos de confiança de 95% (IC95%). Foi considerado estatisticamente significativo um p-valor<0,05.

## Resultados

Participaram do estudo 207 mulheres cisgênero (85,9%), e todas as amostras auto coletadas foram válidas para o teste do HPV (dados não apresentados em tabelas). A média (DP) de idade encontrada foi 48,7 (10,7) anos. Em 125 amostras (60,4%), foi registrada infecção por HPV de alto risco. Em relação às variáveis sociodemográficas e comportamentais (Tabela 1), a maior prevalência do HPV foi em mulheres de 18-29 anos (78,6%), com renda familiar de 1 a 3 salários mínimos (69,5%), usuárias de preservativo (68,5%).

**Tabela 1:** Prevalência (P) e intervalos de confiança de 95% (IC95%) de HPV de alto risco em mulheres vivendo com HIV/aids, segundo características sociodemográficas, comportamentais e clínicas. Vitória, maio de 2021 a maio de 2022 (n=207)

Variável	n (%)	HPV de alto risco	
		P (IC95%)	p-valor^a^
**Faixa etária** (anos)			0,047
18-29	14 (6,8)	78,6 (50,4; 93,0)	
30-39	38 (18,4)	76,3 (60,3; 87,2)	
40-49	64 (30,9)	54,7 (42,4; 66,4)	
50-64	91 (44,0)	54,9 (44,6; 64,9)	
**Escolaridade** (anos)			0,914
0-4	43 (20,8)	55,8 (7,6; 69,8)	
5-8	53 (25,6)	62,3 (48,6; 74,3)	
9-11	92 (44,4)	60,9 (50,5; 70,3)	
≥12	19 (9,2)	63,2 (40,2; 81,4)	
**Raça/cor da pele**			0,460
Branca	66 (31,9)	60,6 (48,4; 71,7)	
Parda	100 (48,3)	57,0 (47,1; 66,4)	
Preta	41 (19,8)	68,3 (52,6; 80,7)	
**Estado civil**			0,871
Solteira	35 (16,9)	57,1 (40,5; 72,3)	
Viúva	40 (19,3)	60,0 (44,3; 73,9)	
Casada	91 (44,0)	59,3 (48,9; 69,0)	
Separada	41 (19,8)	65,9 (50,2; 78,7)	
**Renda familiar** (**salários mínimos**)			0,039
≤1	80 (42,1)	53,8 (42,8; 64,4)	
>1 ≤3	82 (43,2)	69,5 (58,7; 78,5)	
>3	28 (14,7)	46,4 (29,1; 64,7)	
**Tabagismo**			0,612
Nunca fumou	114 (55,3)	63,2 (53,9; 71,5)	
Ex-fumante	61 (29,6)	55,7 (43,1; 67,7)	
Fumante atual	31 (15,1)	58,1 (40,3; 73,9)	
**Frequência de uso de álcool no último mês**			0,220
Nunca	115 (55,8)	40,0 (53,4; 71,0)	
Menos de uma vez por semana	49 (23,8)	49,0 (35,3; 62,8)	
Ao menos uma vez por semana	37 (18,0)	70,3 (53,8; 82,8)	
Todos os dias	5 (2,4)	60,0 (19,9; 90,1)	
**Usa drogas ilícitas**			0,219
Não	178 (86,0)	58,4 (51,0; 65,5)	
Sim	29 (14,0)	72,4 (53,6; 85,6)	
**Histórico de gravidez**			0,960
Não	23 (11,1)	60,9 (40,1; 78,3)	
Sim	184 (88,9)	60,3 (53,0; 67,2)	
**Número de gestações em grupos**			0,658
0 a 4	183 (88,4)	59,6 (52,3; 66,5)	
5 ou mais	24 (11,6)	66,7 (46,0; 82,4)	
**Idade da primeira relação sexual** (anos)			0,298
<15	44 (21,4)	58,6 (50,9; 66,0)	
≥15	162 (78,6)	68,2 (53,1; 80,2)	
**Parceiros sexuais na vida**			0,585
1-2	61 (29,5)	57,4 (44,7; 69,2)	
3-4	56 (27,1)	66,1 (52,7; 77,3)	
≥5	90 (43,5)	58,9 (48,4; 68,6)	
**Parceiros sexuais nos últimos 12 meses**			0,737
0	62 (30,2)	58,1 (45,5; 69,7)	
1	127 (62,0)	60,6 (51,8; 68,8)	
≥2	16 (7,8)	68,8 (43,2; 86,4)	
**Vacina HPV**			0,767
Não	195 (94,2)	60,0 (52,9; 66,7)	
Sim	12 (5,8)	66,7 (37,4; 87,0)	
**Relação sexual anal**			0,885
Não	122 (59,2)	61,5 (52,5; 69,7)	
Sim	84 (40,8)	59,5 (48,7; 69,5)	
**Uso de preservativo**			0,045
Não	115 (55,6)	53,9 (44,7; 62,8)	
Sim	92 (44,4)	68,5 (58,3; 77,2)	
**Citopatologia cervical alterada**			0,220
Não	190 (91,8)	58,9 (51,8; 65,8)	
Sim	17 (8,2)	76,5 (51,3; 90,9)	
CD4^b^			0,858
≤499	40 (19,3)	59,9 (52,2; 67,1)	
≥500	167 (80,7)	62,5 (46,7; 76,0)	
**Carga viral do HIV**			
<20 indetectável	190 (91,8)	58,4 (51,2; 65,3)	0,070
≥20 detectável	17 (8,2)	82,4 (57,1; 94,2)	
**Usa inibidor de protease**			0,669
Não	94 (45,4)	58,5 (48,3; 68,1)	
Sim	113 (54,6)	61,9 (52,6; 70,5)	
**Tempo de uso de terapia antirretroviral** (anos)			0,220
0 a 4	28 (13,5)	58,7 (51,3; 65,7)	
>4	179 (86,5)	71,4 (52,3; 85,1)	
**Diagnóstico de infecção sexualmente transmissível dado pelo médico^c^**			
Não	179 (92,7)	60,9 (53,5; 67,8)	0,802
Sim	14 (7,3)	64,3 (37,5; 84,4)	

^a^p-valor corresponde ao teste exato de Fisher para variáveis dicotômicas e qui-quadrado para variáveis politômicas; ^b^CD4: aglomerado de diferenciação 4; ^c^O diagnóstico de infecção sexualmente transmissível dado pelo médico é a variável com maior número de dados faltantes (7%).

Em relação aos fatores clínicos (Tabela 1), a maior prevalência do HPV de alto risco se verificou naquelas participantes com citopatologia cervical alterada (76,5%, IC95% 51,3; 90,9) e carga viral detectável do HIV/aids com ≥20 cópias/mL (82,4%, IC95% 57,1; 94,2). 

Na análise ajustada (Tabela 2), observou-se que apenas a carga viral do HIV/aids permaneceu significativamente associada à infecção pelo HPV de alto risco. Mulheres com carga viral detectável (≥20 cópias/mL) apresentaram a prevalência de infecção por HPV de alto risco 37,0% maior em comparação àquelas com carga viral indetectável (<20 cópias/mL) (RP 1,37; IC95% 1,00; 1,88; p-valor 0,046). As outras variáveis (faixa etária, renda familiar e uso de preservativo) mostraram tendências de associação, mas sem significância estatística dentro do IC95%. 

**Tabela 2:** Razões de prevalência (RP) ajustadas e intervalos de confiança de 95% (IC95%) para associações do HPV de alto risco em mulheres vivendo com HIV/aids com testes válidos. Vitória, maio de 2021 a maio de 2022 (n=207)

Variável	HPV de alto risco	p-valor^a^
	RP (IC 95%)	
**Faixa etária** (anos)		0,332
18-29	1,18 (0,78; 1,80)	
30-39	1,26 (0,93; 1,69)	
40-49	0,97 (0,72; 1,30)	
50-64	Ref.	
**Renda familiar** (**salários mínimos**)		0,071
≤1	1,10 (0,70; 1,72)	
>1 ≤3	1,40 (0,72; 1,30)	
>3	Ref.	
**Uso de preservativo**		0,068
Não	Ref.	
Sim	1,24 (0,98; 1,56)	
**Carga viral do HIV**		0,046
<20 indetectável	Ref.	
≥20 detectável	1,37 (1,00; 1,88)	

^a^Os valores de p correspondem ao teste z da regressão de Poisson com variância robusta.

Ao se analisar a prevalência da citopatologia cervical, constatou-se o percentual de 8,2% (17/207) de anormalidades. Entre estas, 29,4% (5/17) corresponderam à lesão intraepitelial de alto grau, 35,3% (6/17) à lesão intraepitelial de baixo grau, e 35,3% (6/17) à de células escamosas atípicas de significado indeterminado (Figura 1A). Entre as amostras com citopatologia cervical normal (190/207), 57,4% testaram positivo para tipos de HPV de alto risco. Nas amostras com citopatologia cervical alterada, o HPV de alto risco foi detectado em 100,0% das lesões intraepiteliais de baixo grau, 50,0% das células escamosas atípicas de significado indeterminado e 80,0% das lesões intraepiteliais de alto grau (Figura 1B). Foram observadas citopatologias cervicais alteradas em três pacientes com HPV não detectável, sendo uma com lesão intraepitelial de alto grau e duas com células escamosas atípicas de significado indeterminado. Quase metade (45,5%) das mulheres com múltiplos genótipos possui dois tipos de HPV de alto risco, enquanto 28,7% delas apresentam apenas um tipo (Figura 1C).

**Figura 1:** Distribuição das anormalidades citopatológicas cervicais (n=17) (A) da presença de HPV de alto risco, segundo o resultado citopatológico (n=207) (B), e do número de genótipos de HPV de alto risco em casos de infecção múltipla (n=101) (C) em mulheres com HPV vivendo com HIV/aids. Vitória, de maio de 2021 a maio de 2022

A análise da distribuição dos tipos do HPV de alto risco (Figura 2) revelou ser o tipo 58 o mais prevalente, presente em 21,7% das amostras, seguido pelos tipos HPV 68 (14,5%) e HPV 52 (10,6%). Os tipos HPV 16 e 18 foram encontrados em 3,9% e 5,8% das amostras.

**Figura 2:** Distribuição dos tipos de HPV de alto risco em mulheres com HIV/aids. Vitória, maio 2021 a maio 2022 (n=207)

A análise de associação entre status do HIV/aids e HPV com anormalidades citológicas (Tabela 3) mostrou que mulheres com infecção por HPV 18 têm cinco vezes mais probabilidade de apresentar anormalidades citopatológicas cervicais, com a prevalência de 33,3% (RP 5,0; IC95% 1,9; 13,1; p-valor 0,001), uma associação estatisticamente significativa. Esse resultado sugere que o HPV 18 é fator de risco importante para anormalidades citológicas. A infecção pelo HPV 58 também foi associada ao risco aumentado de alterações citológicas (RP 2,5; IC95% 1,0; 6,3; p-valor 0,05), embora os resultados indiquem a tendência de associação que pode ter sido influenciada pelo tamanho da amostra. Não foi encontrada associação com o HPV 16. Outras variáveis não se mostraram associadas à citopatologia cervical alterada.

**Tabela 3:** Razões de prevalência (RP) e intervalos de confiança (IC95%) para associação entre status do HIV/aids e HPV com anormalidades citopatológicas. Vitória, maio de 2021 a maio de 2022

Variáveis	Citopatologia cervical alterada		p-valor^a^
	Prevalência (%)	RP (IC95%)	
CD4^b^			0,856
≤499	8,4	Ref.	
≥500	7,5	0,9(0,3; 3,0)	
**Carga viral do HIV**			0,136
<20	7,4	Ref.	
≥20	17,7	2,4(0,8; 7,5)	
**Usa inibidor de protease**			0,179
Não	5,3	Ref.	
Sim	10,6	2,0(0,7; 5.5)	
**Tempo de uso de terapia antirretroviral** (anos)			0,207
0a4	14,3	2,0(0,7; 5,6)	
>4	7,3	Ref.	
HPV16			0,648
Não	8,0	Ref.	
Sim	12,5	1,6(0,2; 10,4)	
HPV18			0,001
Não	6,7	Ref.	
Sim	33,3	5,0(1,9; 13,1)	
HPV58			0,050
Não	6,2	Ref.	
Sim	15,6	2,5(1,0; 6,3)	
**Múltiplo genótipo**			0,073
Não	4,7	Ref.	
Sim	11,9	2,5(0,9; 6,9)	

^a^p-valor teste Wald;^b^CD4: aglomerado de diferenciação 4.

## Discussão

Os achados deste estudo indicaram que mulheres vivendo com HIV/aids apresentam alta prevalência de infecção pelo HPV de alto risco, sobretudo por genótipos não incluídos na vacina atualmente disponibilizada pelo Sistema Único de Saúde. Essa suscetibilidade foi associada à carga viral detectável do HIV, demonstrando a importância do uso de TARV na manutenção da carga viral suprimida do HIV para diminuir a prevalência do HPV de alto risco. Os resultados reforçam que a validação integral das amostras obtidas por autocoleta vaginal para o teste PCR-HPV demonstra a eficácia e a viabilidade dessa estratégia como método de rastreamento primário, contribuindo tanto para o diagnóstico precoce de lesões pré-neoplásicas do colo uterino como na identificação dos genótipos de HPV de alto risco.

Entre as limitações deste estudo, destacou-se o delineamento transversal, que impossibilita o estabelecimento de relações causais entre as variáveis analisadas. Além disso, pode haver viés de informação relacionado à exposição a determinados fatores de risco, como o uso de preservativos e o número de parceiros sexuais, uma vez que esses dados foram obtidos por meio de autorrelato. A abordagem diagnóstica também apresenta restrições, visto que se baseou exclusivamente na citopatologia cervical realizada no último ano, sendo a colposcopia indicada apenas nos casos com alterações citológicas. Essa estratégia pode ter levado à subestimação de lesões em mulheres com exames citológicos normais. Por sua vez, a qualidade dos dados laboratoriais foi aprimorada pela verificação dos registros de contagem de linfócitos T CD4+ e carga viral do HIV no sistema eletrônico nacional, além da checagem individual das carteiras de vacinação.

A alta prevalência de infecção pelo HPV de alto risco observada no estudo corrobora achados do estudo nacional realizado em 2023 (10), que reportou a prevalência de 53,8%, variando de 37,0% em São Paulo a 67,3% em Manaus, e outros estudos regionais (12-14). Em nível global, a prevalência de HPV oncogênico em mulheres vivendo com HIV/aids com citopatologia cervical normal é de 48,4%, contrastando com 17,0% em mulheres soronegativas, o que reflete o impacto negativo da infecção pelo HIV/aids na infecção do HPV (15). Uma revisão sistemática realizada em 2020 (16) relatou a prevalência média de HPV oncogênico de 51,0% em países em desenvolvimento, com variações significativas: Benim apresentou prevalência de 87,5% (17), e as Bahamas, de 78,0% (18). Essas elevadas prevalências podem refletir desigualdades sociais no acesso a cuidados de saúde e a maior prevalência de imunossupressão pelo HIV/aids nessas regiões (16,19,20). Prevalências mais baixas foram observadas na Índia (34,7%) (21) e na Etiópia (35,2%) (22), o que pode estar relacionado a fatores locais, como variações no perfil sociodemográfico e estratégias de saúde pública.

A imunossupressão decorrente da infecção pelo HIV compromete a resposta imune celular, dificultando a depuração do HPV, favorecendo a reativação de infecções latentes e contribuindo para a persistência viral (23,24). Neste estudo, a carga viral detectável do HIV foi identificada como o principal fator de risco associado à infecção por HPV oncogênico, aumentando em até 37,0% a probabilidade de infecção. Esse achado está em consonância com evidências provenientes de populações semelhantes em Gana (25), Etiópia (22) e na Bahia (26). Tal associação pode estar relacionada à baixa adesão das pacientes à TARV, o que resulta na manutenção de carga viral detectável e, consequentemente, favorece a expressão das oncoproteínas E6 e E7 do HPV. Esse processo pode ser mediado pela ação das proteínas Tat do HIV, que induzem a liberação de quimiocinas capazes de exacerbar a atividade oncogênica do vírus HPV (7).

Diante disso, torna-se evidente que, embora a TARV seja eficaz na supressão da carga viral do HIV e na redução da prevalência do HPV de alto risco, seu impacto é significativamente potencializado quando iniciada precocemente, preferencialmente com contagem de CD4 acima de 500 células/mm^3^, e mantida com adesão rigorosa (27). Estudos conduzidos nas Bahamas (18) e na região amazônica brasileira (28) reforçam essa observação, ao identificarem maior prevalência de infecção por HPV em mulheres com imunossupressão mais severa, caracterizada por contagem de CD4 inferior a 200 células/mm^3^. Esses dados reforçam a importância do diagnóstico precoce do HIV com tratamento imediato e adesão contínua à TARV como medidas importantes para mitigar a persistência do HPV e suas complicações associadas.

Identificar os genótipos de HPV de alto risco é essencial para aprimorar medidas de prevenção primária e secundária, visando diminuir a incidência e mortalidade de cânceres relacionados ao HPV e fortalecer os programas de vacinação. O teste de RT-PCR para HPV como método de rastreamento primário do câncer de colo do útero é crucial para identificar esses genótipos de alto risco. Neste estudo, todas as amostras autocoletadas foram consideradas válidas para o teste do RT-PCR para HPV, alinhando-se a estudo brasileiro publicado em 2023 (10).

A prevalência de outros tipos de HPV não 16 e 18 é maior na população com HIV/aids, quando comparado com suas contrapartes (15-18,21). Neste estudo, o tipo 58 foi o mais prevalente (21,7%), seguido pelos tipos 68 (14,5%) e 52 (10,6%). Resultados semelhantes foram encontrados no Rio de janeiro (12), na África Ocidental (17) e na Tanzânia (29). Também na América Latina e Caribe (30), assim como na China, em 2023 (31), pesquisas identificaram o tipo 58 como o segundo mais prevalente. Na China, o tipo 58 foi a terceira causa de câncer de colo uterino.

No Brasil, tem sido descrita alta prevalência de HPV oncogênico não 16 e 18 (10,12), o que está de acordo com os achado. No entanto, estudos prévios realizados no Espírito Santo identificaram o tipo 16 o mais prevalente, seguido de outros tipos oncogênicos, como o 33, o 31 e o 51 (14,32).

Embora o HPV 18 não tenha sido o genótipo mais prevalente nesta amostra, ele esteve associado à probabilidade cinco vezes maior de alterações citopatológicas cervicais, destacando-se sua alta capacidade oncogênica (15). Entretanto, o subtipo 58 apresentou tendência de associação com anormalidades citológicas, o que corrobora os achados encontrados na China em 2023 (31) e ressalta a importância de monitorar outros genótipos não 16 e 18 em mulheres vivendo com HIV/aids. Tal abordagem pode subsidiar estratégias de prevenção mais abrangentes e eficazes para direcionar medidas de intervenção nessa população vulnerável. A ausência de associação com o HPV 16 nesta análise pode ser explicada pelo limitado poder amostral deste estudo.

Apesar da eficácia da vacina quadrivalente contra os tipos HPV 16,^18^, 6 e 11, outros genótipos oncogênicos, como HPV58 e 52, estão emergindo especialmente em populações imunocomprometidas (15,31). A população deste estudo apresentou baixa cobertura vacinal (5,8%), porém com 100,0% de eficácia contra os tipos-alvo da vacina quadrivalente utilizada. Entretanto, 75% das vacinadas apresentavam outros tipos oncogênicos, como HPV 58, 52, 68 e 82, o que destaca a necessidade de vigilância contínua e possivelmente vacinas com maior cobertura genotípica, conforme observado por estudos no Brasil (33,34) e em outros países (31).

O estudo revelou alta prevalência de HPV de alto risco em mulheres cisgênero vivendo com HIV/aids, com predominância do genótipo 58 e associação com carga viral detectável do HIV. A autocoleta vaginal, analisada por RT-PCR, demonstrou elevada validade, destacando-se como estratégia eficaz para o rastreamento do HPV no âmbito da saúde pública. Os achados reforçam a necessidade de ações integradas, incluindo-se o início precoce e a adesão sustentada à TARV, a ampliação do rastreamento com teste primário para HPV e a utilização da vacina nonavalente nos programas públicos de imunização, com o objetivo de reduzir a carga da coinfecção HPV/HIV e suas complicações.
